# Sex and Gender Differences in Kidney Transplantation

**DOI:** 10.1016/j.semnephrol.2022.04.011

**Published:** 2022-03

**Authors:** Goni Katz-Greenberg, Silvi Shah

**Affiliations:** 1 Division of Nephrology, Department of Medicine, Duke University Medical Center, Durham, NC; 2 Division of Nephrology, Department of Internal Medicine, University of Cincinnati, Cincinnati, OH

**Keywords:** Sex, gender, men, women, kidney, transplantation

## Abstract

Sex and gender often are used interchangeably, but are two distinct entities, with *sex* being the biological attribute and *gender* including the social, psychological, and cultural aspects of one’s identity. Kidney transplantation has been proven to be the best treatment for end-stage kidney disease, improving both quality of life and life-expectancy for most patients. However, gender disparities in access to and outcomes of kidney transplantation remain despite the plethora of evidence showing the advantages of kidney transplantation to our patients. Data have shown that women are less likely to be waitlisted for a kidney transplant and to receive a deceased donor or a living donor kidney. On the other hand, women are more likely than men to become living kidney donors. Although some state the latter is the result of the female gender to nurture and care for loved ones, others believe this observation is because women often are incompatible with their spouse or child because pregnancy is a strong sensitizing event, which stems from the biological rather than the social differences between the sexes. Influence of sex and gender is not limited to access to kidney transplantation, but rather exist in other areas of transplant medicine, such as the difference observed in transplant outcomes between the sexes, variability in immunosuppression metabolism, and even in more contemporary areas such as recent data showing sex-based differences in outcomes of kidney transplant recipients with coronavirus disease-2019, with males having an increased incidence of acute kidney injury and death.

Kidney transplantation has been proven to be the best form of kidney replacement therapy for patients with end-stage kidney disease (ESKD). Kidney transplantation offers improved quality of life, a survival benefit, and lower cost when compared with chronic dialysis.^1^ In 2018, an all-time high of 22,393 kidney transplants were performed in the United States, with almost 80,000 patients remaining on the waiting list at year-end.^2^ However, not all kidney transplantations are created equal, and racial and gender disparities remain prevalent when assessing differences in access and outcomes of kidney transplantations.^3–6^

Sex is the biological attribute, which includes genetic, anatomic, and endocrine traits, whereas gender includes the social, psychological, and cultural aspects of one’s identity.^7,8^ Although sometimes used interchangeably, sex and gender should be regarded as two conceptually distinct entities, and each may affect patients’ choices and outcomes. Historically, there was no recognition of biological differences between male and female either in human studies or in animal models. In addition, clinical trials, which drive evidence-based medicine, were based mainly on male-sex attributes because women were mostly excluded from scientific research.^9^ In recent decades there has been increasing realization that sex and gender are important determinants of kidney disease progression, referral to transplantation, response to treatment, and patients’ outcomes.^10^

Women have a higher prevalence of chronic kidney disease (CKD) when compared with men; however, the incidence of ESKD is 1.5 times higher in men than women.^2^ Data have shown that women are less likely than men to be deceased or live donor kidney transplant recipients, but more likely to be living kidney donors.^11,12^ Furthermore, women are less likely to be listed on the deceased donor kidney transplant waiting list, and when they are they wait longer from the time of chronic dialysis initiation to active listing.^2^ Several studies have shown that the sex of the kidney transplant recipient affects the pharmacokinetics and/or pharmacodynamics of several commonly used immunosuppressive drugs.^13^ This article reviews the effects of sex and gender on all aspects relevant to kidney transplantation. Figure 1 illustrates some of these differences.

## SEX AND GENDER DIFFERENCES IN CKD AND ESKD

**Figure 2.**
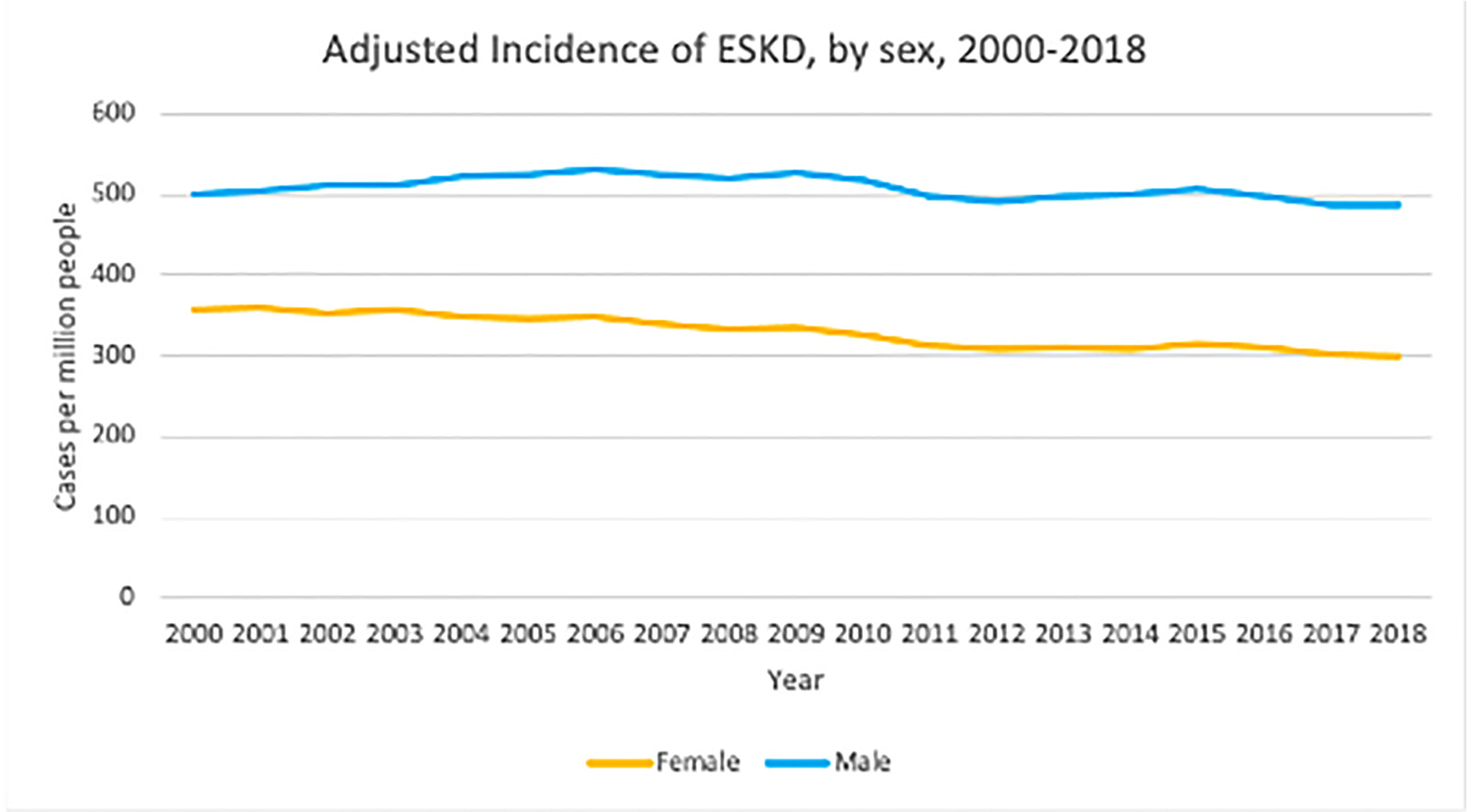
Adjusted end-stage kidney disease (ESKD) incidence in the United States, stratified by sex. Adapted from the US Renal Data System 2020 Annual Data Report.^14^

Both basic and clinical research have shown there is a difference between men and women in the progression of kidney disease.^13^ Women have a higher prevalence of CKD stages 3 and 4, but not of CKD stage 5. For example, in the years 2015 to 2018, 7.33% of adult American women in the general population versus 5.47% of adult men had CKD stage 3, 0.45% of women versus 0.3% of men had CKD stage 4, but men had a higher prevalence of CKD stage 5 (0.07% of women versus 0.15% of men). In addition, men have always had a higher incidence of ESKD when compared with women, and the relative difference continues to increase through the years. Adjusted ESKD incidence was 56% higher among male patients in 2008 and 63% higher in 2018 (Fig. 2).^2^

The etiology of CKD also differs between the sexes; whereas diabetes and hypertension are more prevalent in male patients, autoimmune diseases, such as systemic lupus erythematosus, are a more common cause of CKD in women.^15^ Although sex-attributes contribute to a more rapid progression of CKD in men,^15^ gender-based social and cultural differences lead to late initiation or lack of kidney replacement therapy in women, including later referral for kidney transplant evaluation.^12,16^

## SEX AND GENDER DIFFERENCES IN ACCESS TO THE KIDNEY TRANSPLANT WAITING LIST

Based on Organ Procurement and Transplantation Network data, as of September 23, 2021, women represented only approximately 38% of all waitlisted kidney transplant candidates (34,478 women listed of a total of 90,230).^17^ This imbalance between the sexes on the waiting list exists across all solid-organ lists except lung transplantation.^7^ Several studies have shown that women with ESKD experience 10% to 20% less access to a kidney transplant than men, even after adjustment for demographic and clinical characteristics.^18–21^ Various reasons for why there are fewer women on the kidney transplant waiting list have been described. Reports have shown that physicians and other health care providers often view women as being frailer than men,^22^ and therefore are less likely to discuss kidney transplantation as a preferred kidney replacement therapy option with women who have advanced CKD or ESKD. In one study, women were 1.45 times as likely not to have had discussions with medical professionals about kidney transplantation as a therapeutic option,^23^ and this disparity was even greater in older women; in participants ages 66 to 75 years, women had 29% less access to kidney transplant than men (relative risk, 0.71; 95% confidence interval [CI], 0.68–0.75; *P* < .001), and women older than age 75 years had 59% less access to transplant than men of the same age (relative risk, 0.41; 95% CI, 0.34–0.50; *P* < .001).^19^ The serious consequences of these disparities can be appreciated further by the report from the same study that women of all ages had similar to improved survival benefit after kidney transplant when compared with men.^19^

It is important to note that men do have a higher incidence of organ failure, which is one of the main reasons for the discrepancy in access to transplant.^24^ In a meta-analysis on nondiabetic kidney disease, male sex was associated with a more rapid decrease in kidney function and worse clinical outcomes.^25^ A different study using Chronic Renal Insufficiency Cohort Study data found that women had a lower risk of CKD progression and death compared with men.^26^

**Figure 3.**
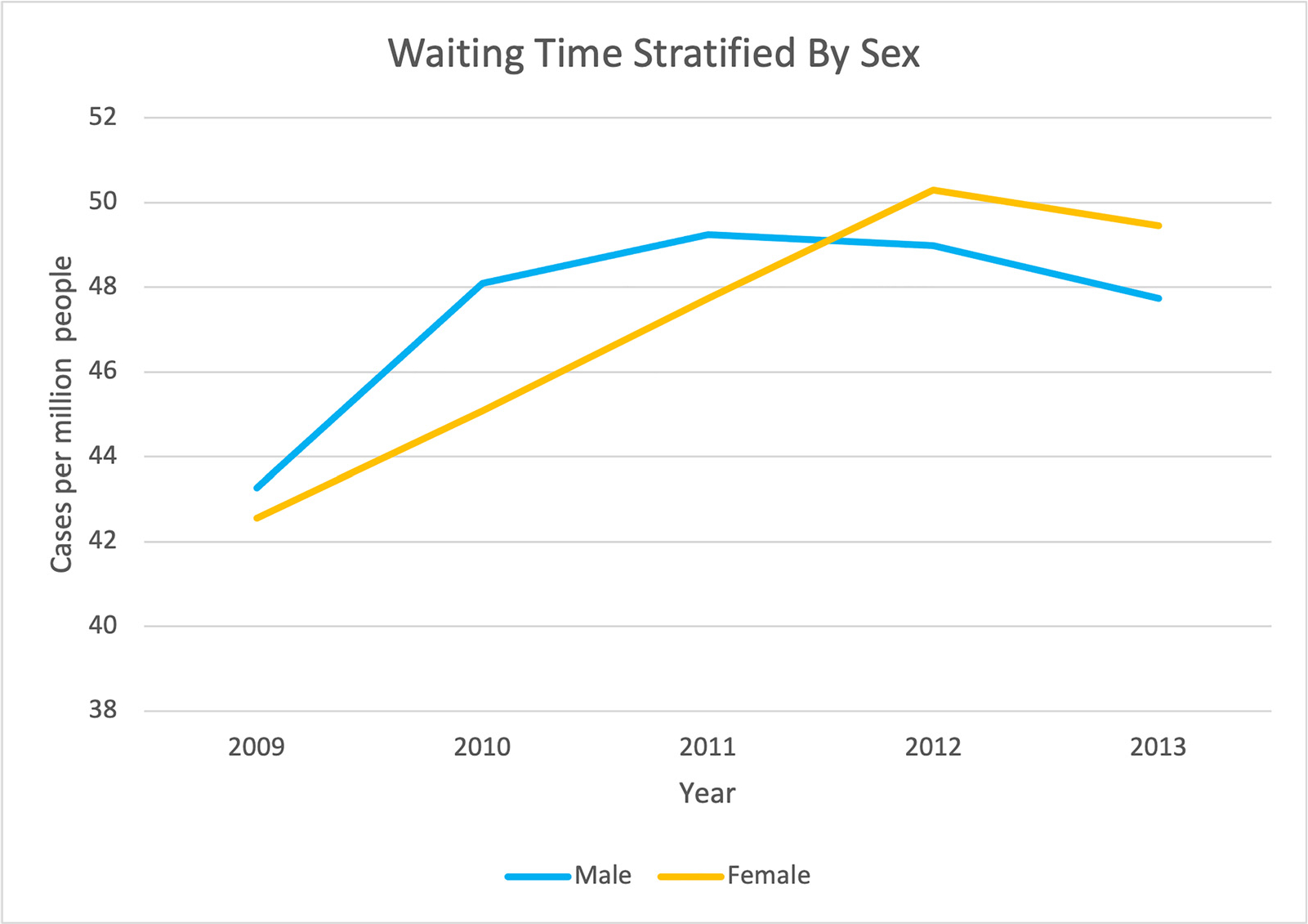
Time on the kidney transplant waitlist in the United States, stratified by sex. Adapted from the US Renal Data System 2020 Annual Data Report.^14^

Although previous studies have shown disparities between the sexes in waitlisting as well as in rates of transplantation,^18,27^ more recent data have shown that the main disparity exists in access to the waitlist.^28^ However, data from the US Renal Data System (USRDS) have shown that in addition to the disparity in access to the waitlist, women wait longer for transplantation; in the past decade, women listed for a transplant in 2013 had a median wait time of 49.4 months, versus 47.7 months for men (Fig. 3).^2^ Even after referral for a kidney transplant evaluation, studies showed that female gender, as well as Black race, older age, and diabetes as the cause of ESKD, all were associated with a lesser likelihood of completing the evaluation,^5^ which prevents these patients from being actively listed on the kidney transplant waiting list, and eventually receiving a kidney transplant. In their study looking at disparities in completion of the transplant evaluation process by race or ethnicity and gender, Monson et al^29^ found that White and Hispanic men completed the medical evaluation faster than White and Hispanic women, whereas there was no difference between Black men and women. Obesity recently was identified as another factor that reduced the likelihood of being listed for a deceased donor kidney transplantation. This was found to be true especially among women, however, obesity did not affect patients’ rate of transplantation once listed.^30^

According to Melk et al, when seen, the sex disparity associated with waitlisting is more affected by socioeconomic factors rather than biological ones.^7^ Interestingly, sex disparities were not found in studies conducted in the United Kingdom^31,32^ or France,^28^ where universal health care exists, as opposed to studies based in the United States. However, a recent report looking into the sex distribution of living kidney donors and recipients in specific countries from across the world, and comparing them with each countries’ general population sex distribution, found that in 10 of 14 countries, including Germany, the United Kingdom, India, and the United States, women were over-represented in the expected donor pool based on the proportion of women within the general population of that country.^33^ Sex disparity also was seen in a study looking at the pediatric population in Europe, where girls were shown to have poorer access to pre-emptive kidney transplants when compared with boys.^34^

Taken together, there is mounting evidence for the existence of disparities in women’s access for evaluation for transplantation, for being listed for transplantation, and in time waiting on the waiting list for transplantation. These trends highlight the importance of continuing to address these differences with well-designed studies focusing on these issues as well as robust collection of national and international data with attention to sex and gender factors.

## SEX AND GENDER DIFFERENCES IN ACCESS TO KIDNEY TRANSPLANTATION

### Sex and Gender in Deceased Donor Kidney Transplantation

The Dialysis Outcomes and Practice Patterns Study showed that fewer women on dialysis received a kidney transplant compared with men, with 5.6% of the women versus 7.0% of the men undergoing a transplantation during the study period (*P* < .05).^35^ Once listed, women also are less likely than men to receive a deceased donor kidney transplant.^15^ One of the main factors, which is believed to contribute to this, is that within all firstcomers, women tend to have higher panel reactive antibodies as a result of the immune reaction associated with pregnancy,^8^ which is an example of how factors associated with the sex of the recipient can lead to unpreventable disparities in rates of kidney transplantation between the sexes. Furthermore, it has been well documented that the prevalence of human leukocyte antigen immunization increases with parity,^36^ which, in turn, may cause greater disparity to women of minorities because they are more likely to have multiple pregnancies.^37^ Interestingly, according to USRDS data, women also are more likely than men to donate their organs when deceased,^38^ with deceased women donors likely to be older and die as a result of cerebrovascular accidents.^7^

### Sex and Gender in Live Donor Kidney Donation

Living donor kidney transplantation is the best treatment for patients with advanced CKD or ESKD.^39^ Kidney donation is considered safe, although recent studies have shown a slightly higher risk of cardiovascular disease and kidney disease in donors than in the general population.^40,41^ In a study meant to assess the cumulative incidence and lifetime risk of ESKD in living kidney donors, kidney donors from the Organ Procurement and Transplantation Network registry were matched to 9,364 participants from the National Health and Nutrition Examination Survey who had no identifiable contraindication to kidney donation and found that donation was associated with an increased risk of ESKD. Furthermore, the cumulative incidence of ESKD per 10,000 at 15 years varied by race and sex, with 96.0 (95% CI, 58.0–158.8) among Black men versus 58.5 (95% CI, 34.2–100.0) among Black women, and 34.0 (95% CI, 22.7–51.0) among White men versus 14.6 (95% CI, 8.8–24.2) among white women (*P* < .001).^42^ Conversely, from a psychosocial aspect, donors were found to have a similar to better quality of life than nondonors,^43^ which is thought to arise in part from their connection with health care, and the realization of the importance of lifelong medical follow-up evaluation.

Women are more likely to become a living donor than they are to receive a living donor kidney donation, and, in fact, women represent 6 of every 10 living kidney donors.^11,25^ Although there is no conclusive evidence why women donate more than they receive,^44^ this inequality likely stems from both sex (biological) and gender (sociocultural) aspects. This higher level of living donation among women is achieved even though women often are incompatible with their spouse or child because pregnancy is a strong sensitizing event. In fact, Bromberger et al^45^ showed that women were incompatible with at least one living donor three times more frequently than men. These findings strongly underscore the willingness of women to be a living donor compared with men.

This remarkable willingness of women to be a living donor probably is related to sociocultural factors,^46^ which in part are related to women’s more traditional role as the caregiver in the family. In fact, studies have shown that women donate first as mothers, followed by as wives or spouses.^47^ However, in her work, Zeiler^48^ raised an ethical concern if the gender disparity seen in living donation is indeed explained by this traditional role of women even in today’s modern society. In their work, however, Rota-Musoll et al^44^ showed that a woman’s decision to donate a kidney was driven mainly by their desire to improve the life and health of the recipient, and also gave them a sense of empowerment, rather than previous studies that showed women donate to their male spouse mainly owing to structural and economic pressures.^49^ Nonetheless, there is a socioeconomic factor that contributes to this disparity because men often are regarded by society as the breadwinners, and if they become living donors it can lead to an economic loss and financial burden for the entire family unit.^11,44,50^

From a biological (sex) standpoint, men have a higher incidence of hypertension and ischemic heart disease, which may preclude them from donation from a medical perspective,^24^ regardless of socioeconomic factors. Despite women donating more, some reports have indicated that women with ESKD are less interested in undergoing a living donor kidney transplantation,^3^ although other reports linked this observation to race rather than gender.^51,52^

### Sex and Gender Differences in Kidney Transplantation Outcomes

When comparing kidney transplant outcome differences between the sexes and the genders, there are inconclusive and conflicting data. Although some studies have shown worse kidney transplant outcomes in women compared with men,^53,54^ others have shown similar outcomes between the sexes,^55^ while others have shown male sex to be an independent prognostic factor for worse outcomes in kidney transplant recipients.^56^

Females are known to produce a more robust cellular and humoral immune reaction than males,^8,57^ driven in part by the effect of sex hormones on immune activation, which can explain the lower allograft survival in women that has been observed in several studies. The heightened immune response can lead to more acute rejection episodes in women, which can lead to allograft injury, and, ultimately, allograft loss. Interestingly, although higher sensitization rates in women are attributed mostly to previous pregnancies, studies in children and young adults also have suggested poorer outcomes in pre-adolescent girls,^58,59^ with no clear explanation for this observation.

On the other hand, studies that have shown an allograft survival advantage in women state better compliance with follow-up evaluation and immunosuppressive medication regimen in women than in men.^24,60^ This observation is believed to stem from the sociocultural differences between men and women, in which women are believed to be more aware of their health state and inclined to follow their health care providers’ recommendations. Differences between males and females also were observed in a preclinical study. In a murine model examining the differences in ischemia-reperfusion injury, female mice had greater tolerance of ischemia-reperfusion injury than male mice. Furthermore, exogenous estrogen administration was found to be protective of ischemia and delayed graft function incidence.^61^

Another factor that has been reported recently to affect the outcomes of women undergoing kidney transplantation is related to the X chromosomes. Biologically, females have two X chromosomes. During fetal development, one copy of the X chromosome will undergo random inactivation, thereby achieving a gene dosage equivalence to males who only have one copy of the X chromosome. This inactivation in the somatic cells is random, and although it is expected that cells will have a 50:50 chance of expressing genes from the maternal copy or paternal copy, it is known that the inactivation can be skewed in as much as 80% of cells, which will display preferential inactivation of maternal or paternal X chromosomes.^62,63^ In a study looking at the skewing of X-chromosome activation as an epigenetic risk factor in kidney transplant outcomes, Simmonds et al^64^ found that female recipients who have X-chromosome skewing are at lesser risk for acute rejection, whereas recipients of female donor kidneys with X-chromosome skewing have a higher risk of allograft failure. Further studies are needed to fully understand the differences in kidney transplantation outcomes between the sexes, and the role the sex chromosomes play.

### Donor-Recipient Sex Mismatch

Sex differences in graft survival are not only associated with the sex of the recipient, but also of that of the donor. There is an increasing body of evidence that the sex of both the donor and the recipient are relevant to the outcome of the kidney allograft. Allografts from female donors have been shown to have higher rates of acute rejection and short-term (1-year) allograft loss.^65^ Puoti et al^24^ showed that female donor kidneys have a worse 5-year survival versus male donor kidneys, while Zeier et al^54^ showed a more specific survival benefit for female recipients of kidneys of male donors compared with male recipients of kidneys of female donors. These observations can be explained by several factors including a lower number of nephrons in the female kidney^66^ and higher expression of human leukocyte antigens, which can cause increased immunogenicity.^24^ However, in a recent report by Lepeytre et al,^67^ when the relationship between recipient sex and graft survival was modified by recipient age and sex, in most recipient age groups (0–14 y, 24–44 y, and ≥45 y), allograft outcomes were poorer for women compared with men only when the donor was male. A similar observation was seen in a study based on USRDS data, in which after adjustment for recipient and donor sex, female recipients of male donor kidneys had worse graft and patient 1-year outcomes compared with all other recipient-donor sex combinations.^55^ One explanation for these findings may be what is known as the H-Y effect. The H-Y antigen system includes seven genes on the Y chromosome that produce amino acid sequences distinct from their X chromosome homologs and have significant tissue expression. They are part of the minor histocompatibility antigens, and their clinical importance has been shown in hematopoietic stem cell transplantation, in which female recipients exposed to H-Y antigens from a male donor had increased risk for graft rejection.^55^ The role of the H-Y antigen system in kidney transplantation is less clear. Following several small studies, Gratwohl et al^68^ were able to show worse outcomes (increased risk of short- and long-term allograft failures and death-censored allograft failures) in female recipients of male donor kidneys.

### Sex Differences in Medications

The biological sex of kidney transplant recipients impacts the pharmacokinetics and pharmacodynamics of several commonly used immunosuppressive drugs.^13^

### Calcineurin Inhibitors

The calcineurin inhibitors tacrolimus and cyclosporine are currently the most widely used immunosuppressive drugs in different combinations. Both drugs are metabolized by the cytochrome P450 enzyme system, by both CYP3A4 and CYP3A5 enzymes, and are substrates of the P-glycoprotein transporter,^69^ which leads to many drug–drug interactions.

Both medications are characterized by high intrapatient variability. Furthermore, sex-related differences in the pharmacokinetics of these drugs have been reported, with studies showing higher weight-normalized clearance in women of both cyclosporine^70^ and tacrolimus.^71^ Many studies have tried to explain this observation without conclusive findings, and sex-specific dosage recommendations are not yet utilized.^13^

### Antimetabolites

Mycophenolic acid is the most used antimetabolite in kidney transplant recipients. Mycophenolic acid is available either as an ester prodrug (mycophenolate mofetil) or as a sodium salt (mycophenolate sodium). Several pharmacokinetics studies have shown that males have approximately 10% to 25% higher clearance of mycophenolic acid.^72,73^ Azathioprine is another antimetabolite drug used in kidney transplantation to prevent rejection. The active metabolite of azathioprine, 6-mercaptopurine, is inactivated by the thiopurine S-methyltransferase enzyme. There is well-documented variability in thiopurine S-methyltransferase activity, which is tested before initiating any patient on this drug. Studies have shown that thiopurine S-methyltransferase expression is 14% higher in men compared with women, and that testosterone increases enzyme activity. However, the clinical significance of these observations have yet to be proven.^74^

### Glucocorticoids

In kidney transplantation, glucocorticoids are used for induction and maintenance immunosuppression as well as for treatment of acute allograft rejection. Biological female sex is associated with lower clearance and increased exposure of the active form: prednisolone.^75^ Not surprisingly, there is also a hormonal influence that has been reported in the literature in which weight-normalized unbound clearance of prednisolone was significantly lower in postmenopausal women compared with premenopausal women.^76^

As shown earlier, the differences in pharmacokinetics and pharmacodynamics of some of the commonly used immunosuppressive agents raises the question of when and how transplant physicians and other health care professionals should consider the sex of the recipient in medication dosing. This may be especially true for drugs for which therapeutic drug monitoring is not performed or available in a clinical practice, such as mycophenolate, prednisone, and azathioprine.

## CONTEMPORARY MATTERS IN KIDNEY TRANSPLANTATION AND GENDER MEDICINE

### Coronavirus Disease-2019 Infection

All kidney transplant recipients are at higher risk for infections than the general population owing to their life-long immunosuppression medication regimens. In the general population, there is a well-established difference between men and women in the susceptibility and severity of infectious diseases, in which men have a higher prevalence for infections and more severe disease.^8,77^ Sex and age differences in immune response are believed to be the reason for the more severe symptoms and higher mortality seen in men compared with women.^78^ This trend also has been seen in data regarding coronavirus disease-2019 (COVID-19) infection, when after controlling for age and comorbidities, men were found to have a 1.5-fold to 2-fold higher mortality risk than women for COVID-19 mortality rates.^79^

Initial studies of COVID-19 infection in kidney transplant recipients did not show a difference in COVID-19 mortality rates between the sexes.^80^ However, a more recent retrospective study that looked at all solid-organ transplant recipients showed that female kidney transplant recipients had a lower risk for acute kidney injury and death compared with male kidney transplant recipients. Interestingly, this observation was not seen in recipients of other solid-organ transplants.^81^ The full effects of the COVID-19 pandemic on kidney transplant recipients remain to be seen.

### Transgender Medicine in Kidney Transplantation

Transgender medicine is an increasingly recognized field in health care. Transgender people are those with a gender identity that is different from the sex assigned to them at birth, whereas cisgender people’s gender identity is the same as their birth-assigned sex. In the United States, the transgender population is estimated to comprise 0.04% to 0.6% of the general population.^82^ Taking into account the increasing prevalence of ESKD in the United States,^2^ it can be estimated that there are approximately 5,000 transgender ESKD patients, with many of them being potential kidney transplant candidates. In addition to challenges faced by all kidney transplant candidates and recipients, the transgender population may have additional challenges such as effects of hormonal therapy, psychosocial aspects, and anatomic changes after gender-affirmation surgery. An example of the latter can be seen with feminizing vaginoplasty and phalloplasty, one of the most common gender-affirming surgeries, which can lead to possible anatomic issues given manipulation of the urethra and risk of subsequent strictures, recurrent urinary tract infections, fistulas, and acute kidney injury.^83^

It is well established that sex hormones play an important role in kidney disease. Low testosterone levels seem to have a negative impact on kidney function. A prospective study found that men with kidney dysfunction and low testosterone levels had an increased risk (2.5 times higher) of all-cause mortality compared with men with normal testosterone levels.^84^ On the other hand, kidney transplantation has been shown to increase sex hormone levels, with testosterone and estradiol levels highest in kidney transplant recipients compared with different stages of CKD, and this observation was associated with a decrease in mortality in genetically male transplant recipients.^85^

For genetically male recipients identifying as women, the increase in estradiol levels after kidney transplantation is important to consider for those seeking gender-affirmation treatment.^86^

Exogenous hormonal therapy can be associated with unique risks, both surgical-specific, and resulting from potential interaction with and effects of common transplant medications. Feminizing drug therapy most commonly includes estrogen in different formulations, an antiandrogen such as spironolactone, as well as progesterone.^87^ A feared adverse effect of estrogen is the association with venous thromboembolism events during surgery,^88^ which can lead to thrombus and allograft loss in the peritransplant phase. Although there is no clear evidence, some opt to temporarily discontinue estrogen to mitigate these risks.^89^ For the transgender person, the risk of venous thromboembolism and graft thrombosis should be weighed against the dysphoria that can accompany cessation of hormonal therapy.

On the other hand, cessation of testosterone supplementation is not necessary given the limited risk of surgical complications.^88^ Exogenous testosterone does have its side effects, which can be more pronounced in the transgender kidney transplant recipient, including alopecia, which can be worsened by tacrolimus and alleviated with finasteride; acne, which can be aggravated by mammalian target of rapamycin inhibitors and corticosteroids; and infectious vaginitis, to which the transplant recipient who underwent female to male gender-affirmation surgery may be predisposed.^83,87^ It is recommended that hemoglobin and hematocrit levels should be monitored closely in patients on exogenous testosterone because it can increase erythropoiesis and therefore increase the risk of post-transplant erythrocytosis.^89^

One of the challenges of the transplant recipient is the need for life-long medication adherence. Previous studies have shown that 20% of all-comer kidney transplant recipients can suffer from depression in the setting of chronic disease, which can be exacerbated further by transplant medications such as calcineurin inhibitors and corticosteroids.^90^ Transgender people undergoing kidney transplantation thus may be at higher risk, with some estimates of up to 70% lifetime risk of different psychiatric comorbidities in this population,^91^ which may partially stem from the stigma and stressors associated with being transgender.

Poorly controlled psychiatric illness is an independent predictor of mortality and could result in nonadherence with medication and follow-up evaluation.^91^ Although all kidney transplant recipients may struggle with medication adherence, this can be especially true in transgender transplant recipients when we consider the side effects of some of the common immunosuppressive agents, and their possible effects on body image. Glucocorticoids, for example, can result in cushingoid changes such as weight gain, while tacrolimus can cause hair loss, and cyclosporine can cause hirsutism.^86,92^ There is a paucity of data on the mental well-being of the transgender transplant recipient population relating to medication adherence and effect of mood disorders. Research is needed to help guide the care and counseling of the transgender patient undergoing transplantation.

### Fertility and Pregnancy in Kidney Transplant Recipients

Fertility is reduced in ESKD. Previous studies have shown a 1:100 probability for a woman with ESKD to have a successful pregnancy, and, more recently, the rate of pregnancy in women undergoing dialysis has been reported to be approximately 18 per 1,000 person-years.^93,94^ After kidney transplantation, fertility is restored in most women within 6 months,^95^ allowing for kidney transplant recipients to become pregnant. However, the risk of pregnancy-related complications, such as worsening kidney function, hypertension, and proteinuria, remains higher in these patients than in the general population.^96,97^ Furthermore, it has been well established that hypertensive disorders of pregnancy, such as preeclampsia, predispose women for CKD later in life,^98^ which can be even more prevalent among kidney transplant recipients. A recent meta-analysis showed that in women with kidney transplants, risk factors for allograft loss after pregnancy include hypertension, proteinuria, preconception graft function, and the interval between kidney transplant and conception.^99^

As such, female kidney transplant recipients who wish to conceive are encouraged to try and become pregnant after a minimum of 1 to 2 years from their kidney transplantation,^96^ as long as they have normal kidney function, no or minimal proteinuria, and no recent rejection episodes.^11^

Another challenge faced by kidney transplant recipients who wish to become pregnant is the known teratogenicity of a few of the immunosuppressive agents. Mycophenolic acid has been associated with an increased incidence of spontaneous abortions and an increase in the incidence of a specific pattern of birth defects.^100^ Women taking mycophenolic acid should undergo counseling before conception, and be offered alternatives to this medication that will be safe for the patient and kidney allograft, as well as minimize fetal risks.^101^

Male fertility also may be low in advanced CKD. Men on dialysis have been found to have impaired spermatogenesis leading to decreased sperm counts and poorly functioning sperm.^102^ In addition, the prevalence of testosterone deficiency ranges between 50% and 75% in men with CKD.^103^ Testosterone deficiency often is unrecognized and, therefore, not treated in this patient population,^104^ and, when it is, there have been conflicting reports regarding the benefit of using testosterone replacement therapy to improve sexual dysfunction in these patients.^105^ Luckily, male fertility usually is restored after kidney transplantation, with the testosterone restored to normal levels, with one study showing normalization in levels of prolactin, luteinizing hormone, and testosterone in male patients who undergo successful kidney transplantation.^105^ Even with the increase in testosterone levels, some patients still experience erectile dysfunction (ED) after kidney transplantation,^106^ with some older data showing as many as 50% of kidney transplant recipients will continue to experience ED.^107^ Factors contributing to ED include medication side effects, anxiety, and change in the endocrine milieu resulting from the transplant.^106^ Evidently, without treatment, ED can impair the quality of life of both the patient and his partner. Although the different treatments for ED are beyond the scope of this review, it is worthwhile to mention sildenafil which has become an important treatment option for these patients.

As in female kidney transplant recipients, the question of the teratogenicity of the different immunosuppressive medications has been studied in children of male recipients. To date, there has been no evidence that the children of male transplant recipients taking mycophenolic acid have an increased proportion of birth defects or other unwanted outcomes compared with the general population.^108^

### Research of Sex and Gender in Transplantation

With the recognition that sex and gender have an important role in assessing disease epidemiology and progression, and response to treatment and patient outcomes, several health research institutes have set out guidelines on how to incorporate these concepts in scientific research. The US National Institutes of Health, for example, requests that researchers provide a plan of how sex will be considered in their study design and data analysis.^109^

Sex and gender are viewed as important concepts in transplantation research, as in other specialties, and should be viewed as two distinct entities, with each having a different influence on the health of the kidney transplant recipient. In their work, Laprise et al,^10^ found that many articles apply the terms sex and gender erroneously or interchangeably. Rarely were sex or gender mentioned in the main objectives of the study, with most studies considering these variables as confounders, and a minority considering them as effect measure modifiers. Laprise et al^10^ provide an example of how members of the female sex may experience increased rates of graft injury resulting from factors such as pregnancy and stronger immune responses, and variability in drug metabolism; whereas there may be lower risk for graft failure in feminine transplant recipients (ie, feminine gender) because of increased adherence to medication and follow-up care. This example shows the complexity of the interaction between sex and gender and underscores the importance of considering both when conducting basic or clinical research. Hopefully, with time and education of the transplant professional community, these concepts will be integrated correctly into transplantation research for the benefit of our patients.

## Figures and Tables

**Figure 1.**
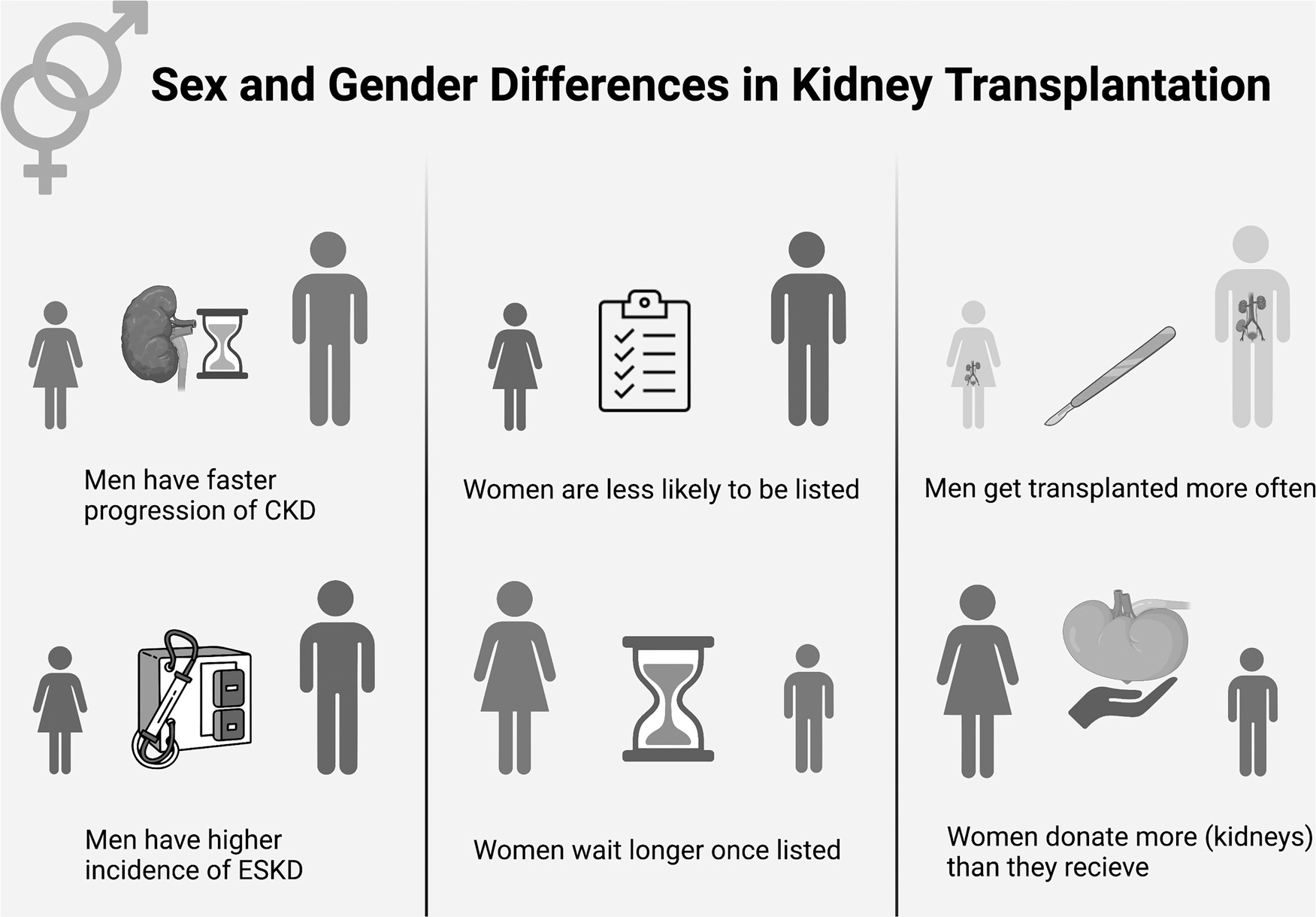
Sex and gender differences in kidney transplantation. Abbreviations: CKD, chronic kidney disease; ESKD, end-stage kidney disease.
